# M^6^A RNA Methylation Mediates NOD1/NF-kB Signaling Activation in the Liver of Piglets Challenged with Lipopolysaccharide

**DOI:** 10.3390/antiox11101954

**Published:** 2022-09-30

**Authors:** Menghui Xu, Ruhao Zhuo, Shengxiang Tao, Yaxu Liang, Chunru Liu, Qingyang Liu, Tian Wang, Xiang Zhong

**Affiliations:** 1 College of Animal Science and Technology, Nanjing Agricultural University, Nanjing 210095, China; 2 College of Veterinary Medicine, Nanjing Agricultural University, Nanjing 210095, China; 3 College of Life Science, Nanjing Agricultural University, Nanjing 210095, China

**Keywords:** m^6^A modification, NOD1, NF-κB, inflammation, immune, oxidative stress, piglet

## Abstract

N^6^-methyladenosine (m^6^A) is the most abundant internal modification that widely participates in various immune and inflammatory responses; however, its regulatory mechanisms in the inflammation of liver induced by lipopolysaccharide in piglets remain largely unknown. In the present study, piglets were intraperitoneally injected with 80 μg/kg LPS or an equal dose of sterile saline. Results indicated that LPS administration increased activities of serum alanine aminotransferase (ALT), induced M1 macrophage polarization and promoted secretion of inflammatory cytokines, and finally led to hepatic lesions in piglets. The NOD1/NF-κB signaling pathway was activated in the livers of the LPS group. Moreover, the total m^6^A level was significantly elevated after LPS treatment. MeRIP-seq showed that 1166 and 1344 transcripts contained m^6^A methylation in control and LPS groups, respectively. The m^6^A methylation sites of these transcripts mainly distributes in the 5′ untranslated region (5′UTR), the coding sequence (CDS), and the 3′ untranslated region (3′UTR). Interestingly, these genes were mostly enriched in the NF-κB signaling pathway, and LPS treatment significantly changed the m^6^A modification in NOD1, RIPK2, NFKBIA, NFKBIB, and TNFAIP3 mRNAs. In addition, knockdown of METTL3 or overexpression of FTO both changed gene levels in the NOD1/NF-κB pathway, suggesting that activation of this pathway was regulated by m^6^A RNA methylation. Moreover, the alteration of m^6^A RNA methylation profile may be associated with the increase of reactive oxygen species (ROS), HIF-1α, and MAT2A. In conclusion, LPS activated the NOD1/NF-κB pathway at post-transcriptional regulation through changing m^6^A RNA methylation, and then promoted the overproduction of proinflammatory cytokines, ultimately resulting in liver inflammation and damage.

## 1. Introduction

The widespread presence of microorganisms in the diet and living environment, including pathogens and non-pathogens, frequently induce immune and oxidative stress in the body, leading to immune dysfunction, metabolism disorder, and even diseases, which indubitably affect the health of animal and human. The liver is rich in immune cells such as macrophages and lymphocytes, and it therefore is considered an important immune organ and plays a vital role in the defense against bacteria and related toxic products, such as lipopolysaccharide (LPS) [1]. As a major component of cytoderms of Gram-negative bacteria, LPS can first be recognized by pattern-recognition receptors (PRRs) resided in liver Kupffer cells, which in turn activate corresponding signal transduction pathways [2] and produce mounts of inflammatory mediators, such as TNF-α, IL-1β, etc. [3]. The overproduction of proinflammatory cytokines inevitably lead to liver lesions, for instance cytoplasm vacuolization, nuclear distortion, and abnormal hepatic lobule structure [4,5], thereby causing growth retardation, morbidity even death of pigs. The nucleotide-binding oligomerization domain (NOD)-like receptor (NLR) family, a prominent class of cytosolic PRRs, mainly include NOD1, NOD2, and NOD-like receptor forming inflammasomes [6,7]. NOD1 is extensively expressed in various parenchymal and nonparenchymal hepatic cells, such as hepatocytes, Kupffer cells, and neutrophils [8,9]. When piglets are exposed to pathogens, NOD1 could sense bacterial products in the host cytosol, activate the nuclear factor κB (NF-κB) and mitogen-activated protein kinase (MAPK) signaling pathways, and stimulate the expression of inflammatory mediators [10,11,12]. In recent years, emerging studies have showed that overexpression of NOD1 is associated with liver damage induced by LPS; however, its mechanisms are still mainly unclear in piglets, particularly on post-transcriptional gene expression mediated by m^6^A RNA methylation.

N^6^-methyladenosine (m^6^A) is the most pervasive internal modification occurring in eukaryotic mRNA [13], affecting RNA biological processes, including splicing, transport, degradation, and translation [14,15,16]. Dynamic and reversible m^6^A RNA methylation is regulated by methyltransferases and demethylases [17]. Methyltransferases, also called “writers”, primarily contain METTL3, METTL14, and Wilms tumor 1-associated protein (WTAP) with the function of methyl installation. The m^6^A modification are removed by demethylases, also known as “erasers”, including FTO and ALKBH5. Additionally, RNA-binding proteins, so-called “readers”, mainly include nuclear YTHDC1, IGF2BP1/2/3, HNRNPC and cytoplasmic YTHDC2, YTHDF1/2/3. These readers are equipped to recognize and bind to m^6^A motif, thereby executing its modification function [18,19,20]. M^6^A RNA methylation plays a critical role in inflammatory response induced by immune stress. It was reported that immunological stress caused by LPS stimulation could affect mRNA m^6^A levels [21,22,23]. Moreover, m^6^A manipulation by knockdown, depletion, or overexpression of methyltransferases, demethylases or “readers” changed the expression of genes associated with inflammation, followed by influencing inflammatory progression. METTL3 deletion increased the expression of MyD88S and inhibited the activation of the NF-κB and MAPK signaling pathways in LPS-stimulated human dental pulp cells (HDPCs) [24]. Silencing FTO inhibited NLRP3 inflammasome-mediated IL-1β expression through FoxO1/NF-κB signaling, thus decreasing macrophage activation [25]. Hou et al. reported that YTHDF2 acted as a “rheostat”, and its reduction triggered inflammation reactions and vascular hyperplasia in hepatocellular carcinoma (HCC) [26]. Nevertheless, the biological importance of m^6^A modification under immune stress and its underlying regulatory mechanisms remain vague in piglets.

Therefore, in the current study, we seek to determine the roles and profiles of m^6^A RNA methylation involved in liver inflammation induced by LPS in piglets, which will provide a new perspective on protecting liver health in inflammatory condition.

## 2. Materials and Methods

### 2.1. Animals and Treatment

All the experimental protocol and procedures were conducted on the basis of the Chinese Guidelines and Animal Welfare and were approved by the Animal Ethics Committee of Nanjing Agricultural University (Permit number SYXK-2017-0027). Twelve male piglets in the growing stages (Duroc × Large White × Landrace; 14.70 ± 0.98 kg BW) were randomly assigned to two treatment groups: the control (CON) group and the Lipopolysaccharide (LPS) group. Then, six piglets in the LPS group received intraperitoneal injection with 80 μg/kg LPS (Sigma-Aldrich, L2880, St. Louis, MO, USA) [27], while the remaining six piglets in the CON group were given an equal dose of sterile saline. At 4 h after injection with LPS or sterile saline, blood samples of piglets were collected by jugular venipuncture. Piglets were sacrificed by the exsanguination after electrical stunning. The liver tissue was immediately harvested and fixed in formalin for follow-up histological analysis. Samples were snap-frozen immediately in liquid nitrogen and stored at −80 °C for further analysis.

### 2.2. Cell Culture

The HepG2 cell line used in the present study was purchased from American Type Culture Collection (ATCC, Manassas, VA, USA). Cells were cultured in DMEM (Gibco, Gaithersburg, MD, USA, 11965) containing 10% fetal bovine serum (Gibco, Gaithersburg, MD, USA) and 1% penicillin/streptomycin (Gibco) and incubated at 37 °C under 5% CO_2_.

### 2.3. siRNA and Plasmid Transfection

Human METTL3 siRNA and control siRNA were synthesized by GenePharma (GenePharma, Shanghai, China) (siMETTL3, 5′-CTGCAAGTATGTTCACTATGA-3′). siRNAs were transfected into HepG2 cells by using Lipofectamine^TM^ RNAiMAX (Invitrogen, Carlsbad, CA, USA). The plasmid including pcDNA-FTO and a pcDNA control vector were transfected into HepG2 cells using Lipofectamine LTX Plus (Invitrogen, Carlsbad, CA, USA) following the manufacturer’s instructions. Cells were transfected at 60–80% confluence and collected 24 h after the transfection.

### 2.4. Serum Biochemical Parameters

Activities of serum alanine aminotransferase (ALT Activity Testing kit, no. C009-2-1) and aspartate aminotransferase (AST Activity Testing kit, no. C010-2-1) were determined by a microplate reader (Thermo Scientific, Wilmington, DE, USA) at 510 nm detection wavelength according to the manufacturer’s instructions (Nanjing Jiancheng Bioengineering Institute, Nanjing, China).

### 2.5. Liver Cytokine Quantification

The concentrations of TNF-α, IL-1β, and IL-10 in the liver and plasma were quantified by using enzyme-linked immunosorbent assay kits purchased from Jiangsu MeiMian Industrial Co., Ltd. (Yancheng, Jiangsu, China). All procedures strictly complied with the manufacturer’s protocol.

### 2.6. Liver Histologic Observation

Harvested liver sections were placed into 4% paraformaldehyde solution for fixation. After soaking for 24 h, those tissues were dehydrated by using a series of ethanol and xylene solutions, and then were embedded in paraffin. After cutting into 5 micron-thick by slicer, the specimen was stained with hematoxylin-eosin (HE). Subsequently, the histomorphology of livers were observed and evaluated by microscope (Nikon ECLIPSE 80i, Nikon Corporation, Tokyo, Japan).

### 2.7. Immunofluorescence

Liver segments of piglets were subjected to immunofluorescence staining. Briefly, six micron-thick slices were obtained from paraffin-embedded livers. Xylene and a series of alcohols were used for section dewaxing. After being soaked in citrate antigen retrieval solution (Beyotime Biotechnology, P0081, Shanghai, China), 0.3% Triton X-100 (Beyotime Biotechnology, ST797), and 3% H2O2-methanol, slices were blocked with 5% BSA for 1–2 h at room temperature. Next, specimens were incubated at 4 °C overnight with rabbit-anti-F4/80 (Servicebio, GB113373, Gent, Belgium) and cultured for 1 h with the secondary antibody, Alexa Fluor Plus 647 goat anti-rabbit IgG (Invitrogen, A32733). After all antigens had been labeled, nuclei were stained with Hoechst 33342 (Invitrogen, H3570). Images were acquired using a confocal laser scanning microscope (Carl Zeiss, Oberkochen, Germany) and analyzed by ImageJ program V1.8.0.112 (National Institutes of Health, Bethesda, MD, USA).

### 2.8. Measurement of ROS

The amount of reactive oxygen species (ROS) in the liver was acquired by dihydroethidium (DHE) staining. Firstly, 5-μm cryosections obtained from snap-frozen liver tissues were stained with ROS dye (Servicebio, Wuhan, China, CAS: GDP1018) and incubated at 37 °C for 30 min in a dark place. Next, the DAPI dye cover sections and stain cell nucleus for 10 min at room temperature in the dark. The portions were sealed by anti-fluorescence quenching sealed tablets after PBS three times washing. Finally, the image of sections was gained under a fluorescence microscope (LSM 700-Zeiss, Zeiss Corporation, Jena, Germany), and the fluorescence level of ROS was assessed by an Image-Pro Plus 6.0 (Media Cybernetics, Rockville, MD, USA) software.

### 2.9. RNA Isolation, Real-Time qPCR

Total RNA was isolated from 50-mg liver samples using TRIZol reagent (TaKaRa, Otsu, Shiga, Japan, CAS: 9108) in accordance with the manufactures’ protocol. The concentration, integrity, and purity of extracted total RNA were quantified by Thermo NanoDrop 2000 Ultra Trace visible spectrophotometer (Thermo Fisher, Waltham, MA, USA). Subsequently, 1 µg of RNA were reverse-transcribed into complementary DNA (cDNA) using the PrimerScript RT reagent kit (TaKaRa, Otsu, Shiga, Japan, CAS: RR036A). With 2-μL diluted complement DNA, real-time quantitative PCR (RT-qPCR) were performed to calculate the expression of target genes utilizing the ABI StepOnePlus^TM^ PCR system. The RT-qPCR thermal profile was as follows: 3 min at 95 °C, 40 cycles of 10 sec at 95 °C, and 30 s at 60 °C. All primers that appeared in this study were showed in Table 1. The relative transcript level of target genes was assessed by the 2^−∆∆Ct^ method after selecting GAPDH as a reference gene.

### 2.10. Western Blot

Total protein of liver was extracted using a radio-immunoprecipitation assay (RIPA) buffer (Beyotime Biotechnology, P0013B) containing inhibitors for proteases and phosphatases. The protein concentrations were determined by BCA Protein Assay kit (Beyotime Biotechnology, P0012). A total of 30-µg proteins was electrophoresed in 4–12% SDS-PAGE gels and then transferred onto immobile membrane (PVDF membrane, Merck Millipore, Darmstadt, Germany, CAS: IPVH00010). The membranes were blocked with 5% non-fat dry milk in Tris-buffered saline Tween-20 buffer (TBST) for 2 h, and incubated at 4 °C overnight with anti-METTL3 (Abcam, Cambridge, UK, ab240595, 1:4000), anti-ALKBH5 (Proteintech, Rosemont, IL, USA, 16837-1-AP, 1:5000), anti-YTHDF2 (Proteintech, 24744-1-AP, 1:10000), anti-NOD1 (ABclonal, Woburn, MA, USA, A1246, 1:1000), anti-RIPK2 (Proteintech, 15366-1-AP, 1:1000), anti-IKB-α (Proteintech, 10268-1-AP, 1:2000), anti-P-p65 (Affinity, West Bridgford, UK, AF2006, 1:1000), anti-HIF-1α (Proteintech, 20960-1-AP, 1:5000), anti-MAT2A (Proteintech, 55309-1-AP, 1:2000), anti-β-actin (Proteintech, 60008-1-Ig). Next, after washing three times with TBST, the membranes were cultured for 90 min at room temperature with appropriate secondary antibodies, including 1:6000-dilution of goat anti-mouse (Abcam, ab205718) and goat anti-rabbit (Abcam, ab205719). The blots were detected with an enhanced chemiluminescence substrate kit (Biosharp, Hefei, China). Finally, images were captured by a luminescence image analyzer LAS-4000 system (Fujifilm Co., Ltd. Tokyo, Japan) and the band density was quantified with ImageJ program V1.8.0.112 (National Institutes of Health, Bethesda, MD, USA). β-actin was used as internal control.

### 2.11. Quantitative Analysis of Total m^6^A

The m^6^A levels of total RNA were assessed by using an EpiQuikTM m^6^A RNA methylation quantification kit (Epigentek; Wuhan, China, CAT. No. p-9005) according to the manufactures’ protocol. Briefly, 200 ng aliquots of RNA were extracted from liver tissues. Negative control, positive control, and RNA samples were bound to strip wells using RNA high binding solution. Then, capture and detect antibodies were added into the mixed solution. After the detected signal was enhanced, the OD intensity of m^6^A was quantified at 450 nm absorbance in a microplate spectrophotometer (Thermo Fisher, Waltham, MA, USA).

### 2.12. RNA-seq and MeRIP-seq

Total RNA was extracted from piglets’ livers with TRIZol reagent (TaKaRa, Otsu, Shiga, Japan, CAS: 9108) in accordance with the manufactures’ protocol, and then polyadenylated RNA was enriched by using PolyATtract^®^ mRNA Isolation System III (Promega). Next, the mRNA was sonicated on ice to yield RNA fragments. Partial mRNA samples were saved as input control and performed RNA-seq. A total of 5 µg fragmented mRNA was incubated with 12 µg anti-m^6^A antibody (Synaptic Systems) in 1 × IP buffer (10 mM Tris-HCl, pH 7.4, 150 mM NaCl, and 0.1% lgepal CA-630) for 2 h at 4 °C. Subsequently, the m^6^A-IP mixture was incubated with prepared protein A beads for additional 2 h at 4 °C on a rotating wheel, and after that, bound mRNA was eluted with 100 µL elution buffer (6.7 mM N^6^-Methyladenosine-5′-monophosphate sodium salt in IP buffer) and precipitated by ethanol and sodium acetate. Finally, both the m^6^A-IP samples and the input samples were carried out first-stand cDNA synthesis, and sequencing then was conducted on Illumina NovaSeq 6000 according to the manufactures’ instructions.

### 2.13. Statistical Analysis

All statistical analysis was performed using the SPSS 25.0 (SPSS Inc, Chicago, IL, USA). Statistical significance was determined using a two-tailed unpaired the Student’s *t* test between two groups. Measurement data were expressed as the mean ± standard error of the mean (SEM), and *p* < 0.05 was considered statistically significant.

## 3. Results

### 3.1. LPS Induced Hepatic Inflammation and Damage in Piglets

**Figure 1:**
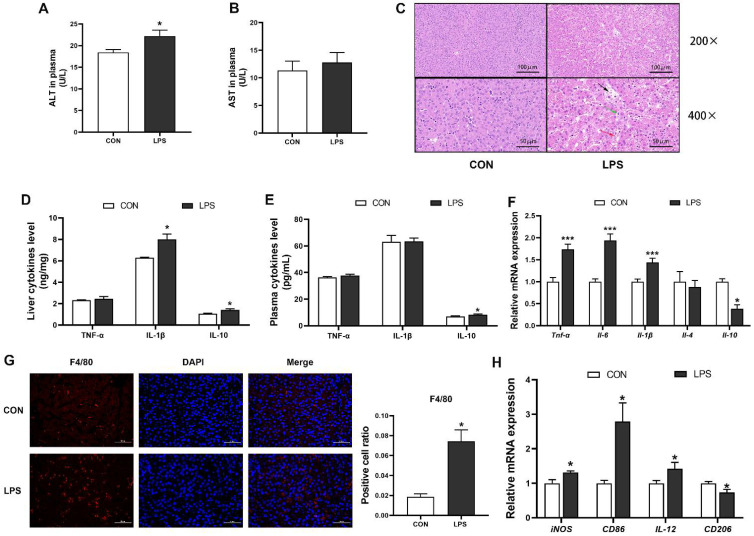
LPS led to inflammation and damage in the liver of piglets. (**A**,**B**) Activities of ALT and AST in plasma of piglets. (**C**) Liver slices were stained with hematoxylin and eosin to assess tissue injury. (H&E, ×200, scale bar = 100 μm; ×400, scale bar = 50 μm). The black arow indicates enlarged intercellular space; The green arow showed damaged cellular structures; The red arow showed blood cells stasis. (**D**,**E**) TNF-α, IL-1β, IL-10 concentrations in the liver and plasm were measured by ELISA. (**F**) The mRNA abundance of TNF-α, IL-6, IL-1β, IL-4, IL-10 were assessed in livers by RT-qPCR. (**G**) The liver sections were subjected to F4/80 immunofluorescence. DAPI was used for nuclear staining and a confocal laser scanning microscope (40× magnification) with Z-scan analysis. Scale bar = 50 μm. The ratio of F4/80-positive cells were quantified by ImageJ analysis. (**H**) the mRNA levels of iNOS, CD86, IL-12 and CD206 were measured in the total liver by RT-qPCR. n = 4−6 per group. Error bars represent the mean ± SEM. * represents significant differences between the CON group and the LPS group; * *p* < 0.05, *** *p*< 0.001.

Following hepatic injury, ALT and AST situated in hepatocyte gain access into peripheral blood by leaking out of the cytoplasm [28]. In the present study, there was a significant increase in the plasma ALT activity in piglets challenged with LPS compared with control piglets (Figure 1A), but no significant different in the plasma AST activity (Figure 1B). Additionally, H&E staining demonstrated that control livers exhibited complete histological structures. On the contrary, damaged structures, enlarged intercellular space and blood cells stasis were found in livers of LPS group (Figure 1C). These morphological results indicated that LPS injection gave rise to liver lesions of piglets. Next, we observed that the concentrations of IL-10 in plasma and IL-1β, IL-10 in liver of the LPS group were significantly higher than those in the CON group (Figure 1D,E). The mRNA expression of proinflammatory cytokines, such as TNF-α, IL-1β, IL-6, was significantly increased in the LPS group (Figure 1F). The anti-inflammatory cytokine, IL-10 mRNA expression showed a marked decrease, but the expression of IL-4 mRNA was not significantly changed in the LPS group (Figure 1F). Moreover, through F4/80 immunostaining analysis, we found that the number of macrophage infiltration was significantly increased in livers of the LPS group (Figure 1G). Moreover, the mRNA expression of M1 macrophage marks, including iNOS, CD86 and IL-12, was obviously elevated after LPS treatment, and with a concomitant decrease in the expression of an M2 macrophage marker, CD206 (Figure 1H). Taken together, these results suggested that LPS administration triggered M1 macrophage polarization and hepatic inflammatory response, followed by causing hepatocyte damage in piglets.

### 3.2. LPS Triggered the Inflammatory Response through the NOD1/NF-κB Signaling Pathway

**Figure 2:**
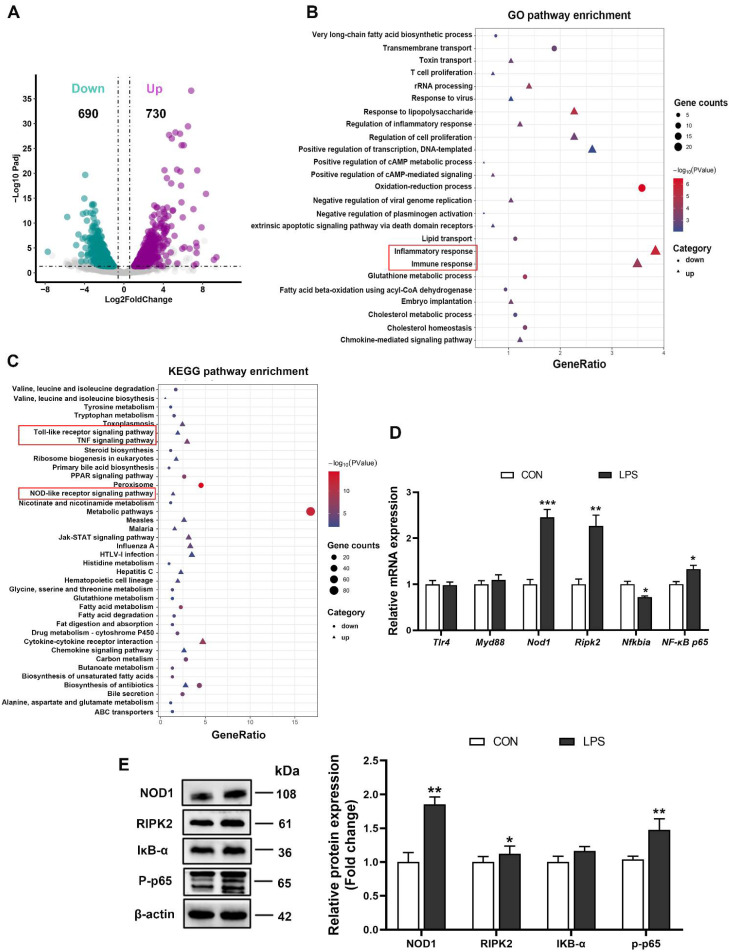
The NOD1/NF-κB signaling pathway was activated in the liver of piglets challenged with LPS. (**A**) Volcano plots of comparative RNA-seq data between the CON group and the LPS group. (**B**) GO analysis based on the RNA-seq in the biological process of differential transcripts. (**C**) Representative KEGG analysis with the transcript corresponding to different genes. (**D**) The mRNA levels of TLR4, MyD88, NOD1, RIPK2, NFKBIA, NF-κB p65 were evaluated by RT-qPCR. (**E**) Hepatic protein levels of NOD1, RIPK2, IκB-α, and P-p65 were assessed by western blot, quantified using ImageJ analysis, and normalized to β-actin. n = 4−6 per group. Results are showed as means with SEM represented by a vertical bar. * represents significant differences between the CON group and the LPS group; * *p* < 0.05, ** *p*< 0.01, *** *p*<0.001.

RNA sequencing (RNA-seq) was employed to identify the transcriptome profiles after LPS stimulation. Results indicated that LPS treatment resulted in an up-regulation of 730 genes and down-regulation of 690 genes in the liver of piglets (Figure 2A). Gene Ontology (GO) analysis showed the differentially expressed genes were significantly enriched in immune and inflammatory responses (Figure 2B). Kyoto Encyclopedia of Genes and Genomes (KEGG) pathway analysis demonstrated that among upregulated genes, the significantly enriched pathways were involved in the ‘TNF signaling pathway’, the ‘Toll-like receptor signaling pathway’, and the ‘NOD-like receptor signaling pathway’ (Figure 2C). Next, we examined the expression levels of key genes in the Toll-like and NOD-like receptor signaling pathways. The results showed that the mRNA levels of NOD1, RIPK2, and NF-dB p65 were significantly increased, and the IκB-α mRNA expression was evidently decreased in the LPS group compared with the CON group, whereas no appreciable difference was noted in TLR4 and MyD88 levels (Figure 2D). Meanwhile, the protein expression of NOD1, RIPK2 and phosphorylated NF-κB p65 was also higher upon LPS injection, although the change of IκB-α protein expression was not significant (Figure 2E). These results indicated that LPS activated the NOD1/NF-κB signaling pathway to induce immune and inflammatory responses.

### 3.3. M^6^A RNA Methylation Profiles of Liver in Piglets after LPS Administration

**Figure 3:**
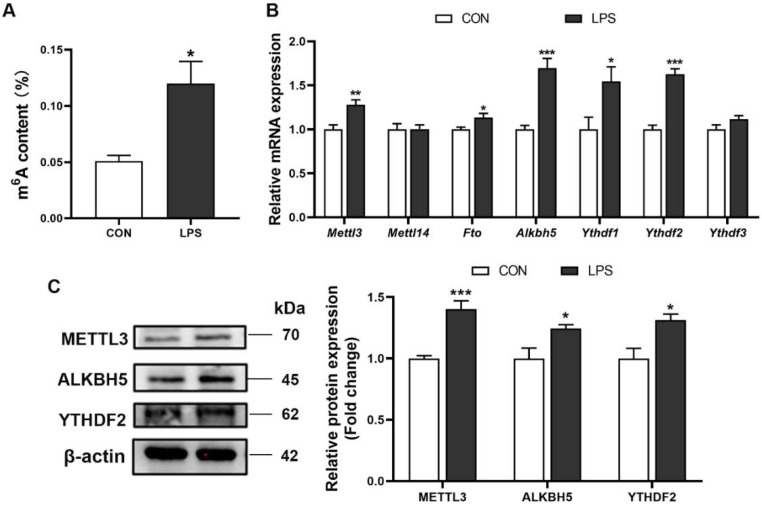
The abundance of m^6^A RNA methylation in the liver of piglets. (**A**) The total m^6^A level was determined by ELISA. (**B**) The mRNA expression of genes related to m^6^A modification were measured by RT-qPCR. (**C**) Hepatic protein levels of METTL3, ALKBH5, and YTHDF2 were evaluated by western blot, and quantification of image density was conducted by ImageJ software. n = 4−6 per group. Results are presented as means with SEM represented by a vertical bar; * represents significant differences between the CON group and the LPS group; * *p* < 0.05, ** *p*< 0.01, *** *p* < 0.001.

**Figure 4:**
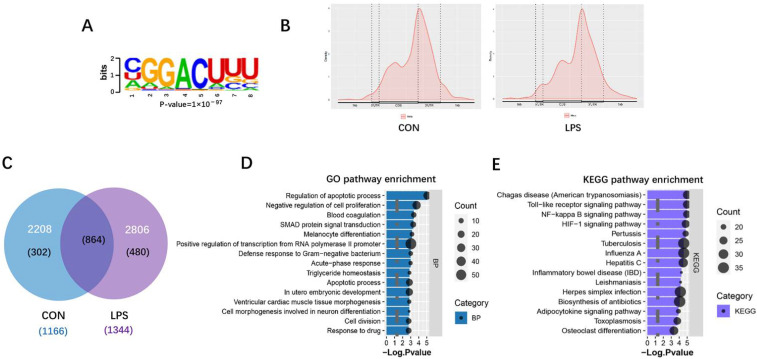
MeRIP-seq of transcriptome in the liver of CON and LPS groups. (**A**) Consensus m^6^A motif of the LPS group, as assessed by MEME. (**B**) Metagene profiles of m^6^A peaks across mRNA transcriptome. (**C**) Venn plot showed the differentially expressed m^6^A peaks and relevant genes in the CON and LPS groups. (**D**,**E**) The top 15 GO and KEEG enrichment analyses of m^6^A-modified genes in the LPS group.

The total m^6^A level and it-related genes and protein expression were measured in the liver of piglets. Results showed that the liver from the LPS group exhibited higher m^6^A modification abundance than that in the control group (Figure 3A). Compared with the CON group, mRNA levels of METTL3, FTO, ALKBH5, YTHDF1, and YTHDF2 displayed significant increase (Figure 3B), and the protein expression of METTL3, ALKBH5, and YTHDF2 were also increased in the LPS group (Figure 3C). Next, to observe transcriptome-wide m^6^A distribution, methylated RNA immunoprecipitation sequencing (MeRIP-seq) was performed. The m^6^A consensus motif (GGACU) was identified by Multiple Em for Motif Elicitation (MEME) (Figure 4A). Metagene plot of m^6^A enrichment across mRNA transcriptome showed that m^6^A peaks primarily enriched in the 5′ terminate (near the start codon), the coding sequence (CDS), and 3′ terminate (near the stop codon) both in control and LPS groups (Figure 4B). In addition, we identified 2208 m^6^A peaks covering 1166 genes in controls and 2806 m^6^A peaks in the LPS group that contained 1344 genes, respectively (Figure 4C). Next, the top 15 GO terms of genes with m^6^A peaks in the LPS group revealed that differential transcripts were clustered in defense response to Gram-negative bacterium and the regulation of apoptotic process and cell proliferation (Figure 4D). Meanwhile, KEGG pathway analysis demonstrated m^6^A-modified genes were significantly enriched in the ‘Toll-like receptor signaling pathway’, the ‘NF-κB signaling pathway’, and the ‘HIF-1 signaling pathway’ (Figure 4E). Collectively, these findings showed m^6^A modification widely distributed on transcripts in inflammatory signaling pathways, which provided the possibility that m^6^A RNA methylation involved in the regulatory mechanism of LPS-induced inflammation.

### 3.4. The NOD1/NF-κB Pathway Was Regulated by m^6^A Methylation Modification

**Figure 5:**
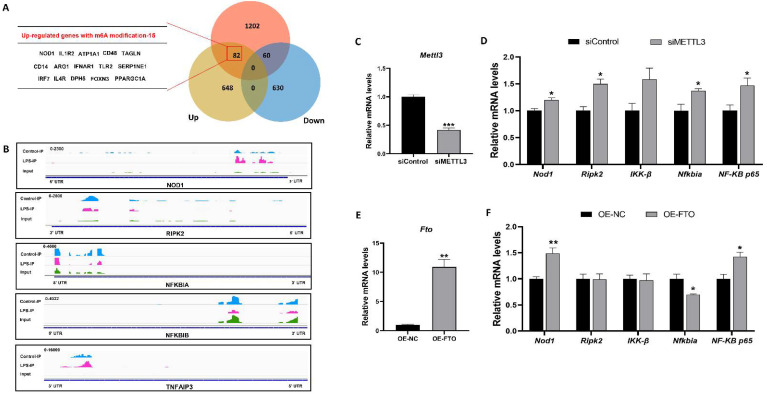
M^6^A RNA methylation modulated the NOD1/NF-κB signaling pathway. (**A**) Venn diagram showed the relationship between m^6^A modification and mRNA expression by the combined analysis of RNA-seq and MeRIP-seq. (**B**) The m^6^A peak profile on NOD1, RIPK2, NFKBIA, NFKBIB, and TNFAIP3 mRNAs were examined by IGV. Wathet blue, dark blue, and red patterning, respectively, represented m^6^A peaks on mRNA in the LPS, CON and input groups. (**C**) The METTL3 mRNA expression in the siControl and siMETTL3 groups. (**D**) Effect of the knockdown of METTL3 on key genes of the NOD1/NF-κB signaling pathway in HepG2 cells. (**E**) The FTO mRNA expression in the OE-NC and OE-FTO groups. (**F**) Effect of the overexpression of FTO on key genes of the NOD1/NF-κB signaling pathway in HepG2 cells. Results are showed as means with SEM represented by a vertical bar (n = 3). * represents significant differences between the CON group and the LPS group; * *p* < 0.05, ** *p*< 0.01, *** *p* < 0.001.

To target m^6^A-modified transcripts, combined analysis of m^6^A-sequence and RNA-sequence was used in this study. As a result, the levels of 142 transcripts marked with m^6^A peaks were significantly changed after LPS treatment, among which 82 gene expression were up-regulated and 60 gene expression were down-regulated (Figure 5A). Notably, among the 82 methylated and upregulated genes, we found the NOD1 and several inflammation-related genes, such as IL1R2, IFNAR1, TLR2, and IL4R. Then, Integrative Genomics Viewer (IGV) plots of m^6^A peaks at mRNAs associated with the NOD1/NF-κB pathway were presented in Figure 5B. Results indicated that the m^6^A methylation sites of NOD1, RIPK2, NFKBIA, NFKBIB, and TNFAIP3 mainly distributed in the 5’ untranslated region (5’UTR), the coding sequence (CDS), and the 3’ untranslated region (3’UTR). Moreover, the m^6^A abundance was higher in NOD1 and TNFAIP3 mRNAs, but less in RIPK2, NFKBIA and NFKBIB mRNAs in the LPS group compared with the CON group. Next, to explore the relationship between m^6^A RNA methylation and the NOD1 signaling pathway, we knocked down METTL3 and overexpressed FTO in HepG2 cells. Results showed that the knockdown of METTL3 significantly increased the expression of NOD1, RIPK2, NFKBIA, and NF-κB p65 mRNA (Figure 5D). The overexpression of FTO remarkably improved the levels of NOD1 and NF-κB p65 mRNA and decreased the NFKBIA mRNA expression (Figure 5F). Based on these findings, we demonstrated that m^6^A RNA methylation modulated the NOD1/NF-κB signaling pathway.

### 3.5. Increase of ROS, HIF-1α and MAT2A May Contribute to Changes of m^6^A Modification

**Figure 6:**
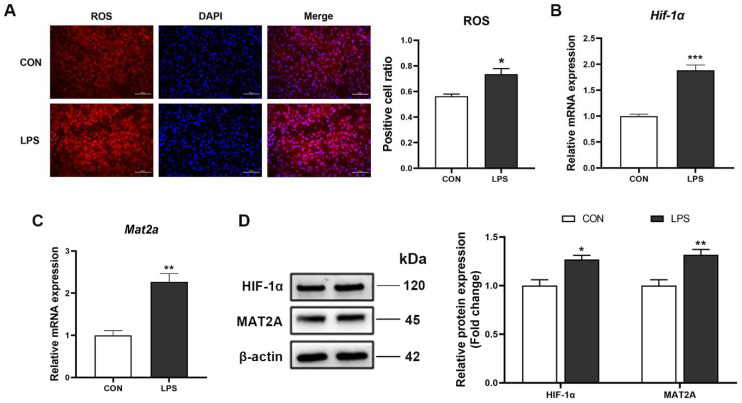
The content of ROS, HIF-1α, and MAT2A in the liver of piglets. (**A**) ROS contents were detected by using dihydroethidium (DHE)-stained liver cryosections in piglets (40× magnification). Scale bar = 50 μm. ImageJ analysis was used to quantify the number of ROS-positive cells. (**B**,**C**) The HIF-1α and MAT2A mRNA levels were examined in the total liver by RT-qPCR. (**D**) HIF-1α and MAT2A protein levels were assessed by western blot, quantified using ImageJ analysis, and normalized to β-actin. n = 4−6 per group. Results are showed as means with SEM represented by a vertical bar. * represents significant differences between the CON group and the LPS group; * *p* < 0.05, ** *p*< 0.01, *** *p* < 0.001.

In the present study, we noticed that ROS content was significantly higher in the LPS group compared with the CON group (Figure 6A), suggesting that LPS treatment resulted in the disorder of antioxidant defense systems in the liver of piglets. Moreover, the expression of hypoxic inducible fator-1 alpha (HIF-1α) was also significantly elevated after LPS treatment (Figure 6B,D). It was reported that HIF-1α could promote the transcription of the methionine adenosyltransferase 2 alpha (MAT2A) enzyme, which in turn catalyze the production of methyl donors and enhance cell methylation levels [29,30]. Therefore, we measured MAT2A mRNA and protein levels. The results indicated that the mRNA and protein of MAT2A were remarkably increased in LPS-activated livers compared with control livers (Figure 6C,D).

## 4. Discussion

Post-transcriptional m^6^A RNA methylation prevalently participates in the modulation of gene expression, immune, and inflammatory responses. However, few investigations uncovered the role and mechanisms of m^6^A RNA methylation in LPS-induced hepatic inflammation in piglets. Here, our present study demonstrated that LPS stimulation increased the total m^6^A level, activated the NOD1/NF-κB pathway, and induced M1 macrophage polarization and expression of proinflammatory cytokines, eventually leading to liver damage in piglets. Importantly, m^6^A RNA methylation could regulate the activation of the NOD1/NF-κB pathway. Therefore, we suggested that LPS activated the NOD1/NF-κB signaling pathway through changing m^6^A RNA methylation, thus inducing inflammation response and liver injury in piglets.

Numerous studies demonstrated that as a member of microbe-associated molecular patterns (MAMPs), LPS was recognized by pattern-recognition receptors (PRRs) located on innate immune cells, which mainly included Toll-like receptors (TLRs) and nucleotide-binding-domain- and leucine-rich-repeat-containing receptors (NLRs) [31,32]. Intracellular NOD1 is a special NLRs that participate in the recognition of pathogenic microorganisms. It has been well-documented that NOD1 is not only activated by bacterial peptidoglycan (PGN), but also is sensitive to the stimulation of LPS [33,34]. In this study, we found that the gene and protein expression of NOD1 were significantly increased after LPS treatment. NOD1 encodes an intercellular multidomain scaffolding protein, which is comprised of a caspase activation and recruitment domain (CARD) and a NOD, and multiple leucine-rich repeats (LRRs). When NOD1 binds to the ligand, it will undergo a conformational change and recruit receptor-interacting serine-threonine kinase 2 (RIPK2) [35], followed by activating downstream NF-κB and MAPK signaling pathways [36]. The activation of NF-κB signaling requires the formation of phosphorylated IκB catalyzed by the inhibitor of nuclear factor-κB (IκB) kinase (IKK) complexes, which promote the release of NF-κB dimers into the nucleus and induce the production of inflammation related genes [37,38]. In our experiment, LPS stimulation upregulated the expression of NOD1, RIPK2 and phosphorylated NF-κB p65 both mRNA and protein levels, suggesting that LPS induced the activation of the NOD1/NF-κB pathway in the liver of piglets. Macrophages play a crucial role in the initiation of innate immune response. Activated macrophages are usually differentiated into two subtypes, an inflammation-promoting M1 macrophages and an anti-inflammatory M2 macrophages [39]. Liu et al. indicated that M1 macrophage markers, such as CD86, iNOS, TNF-α, showed significantly higher expression in LPS-stimulated RAW264.7 cells [40]. In this study, we observed that M1 macrophage markers, iNOS and CD40, significantly increased, and the M2 macrophage marker, CD206, significantly decreased after LPS injection. Moreover, the proinflammatory cytokines expression including TNF-α, IL-1β, IL-6, produced by M1 polarization were also higher in the LPS group, implying that LPS elicited immune and inflammatory responses in the liver of piglets. This evidence suggested that LPS treatment in piglets activated the NOD1/NF-κB signaling pathway and caused hepatic inflammation and injury.

Accumulating evidence have reported that the alteration of m^6^A modification is a widespread phenomenon under stress conditions. In the current study, the change of total m^6^A abundance was consistent with previous reports, in that RNA m^6^A levels were significantly increased in livers treated with LPS [22,41]. However, up to now, the reasons of m^6^A methylation variation caused by LPS in livers remained elusive. In the current study, we observed that ROS content was significantly elevated, with concomitant increase in m^6^A methylation modification in the liver after LPS administration, which was consistent with our previous studies in mice and HepG2 cells [14,42]. These also supported that ROS could regulate major epigenetic processes [43,44]. Hence, we postulated the change of m^6^A methylation modification may be related to the increase in ROS content induced by LPS. Interestingly, it has been reported that ROS can promote Hypoxia-inducible factor 1 alpha (HIF-1a) transcription by activating the HIF-1a promoter or regulating hydroxylase function [45,46]. In this study, HIF-1α levels were also significantly increased in the LPS group. As a transcription factor, HIF-1α can coordinate transcriptional programs and regulate gene expression [47]. Liu et al. reported that HIF-1α could bind to relevant sites in the promoter region of MAT2A and accelerate its transcription [30]. MAT2A could catalyze the generation of S-adenosylmethionine (SAM), which is the most important methyl group donor for most of the methylation reactions [48,49]. Bedi et al. reported that METTL3 could catalyze the transfer of a methyl group from the cofactor SAM to the N^6^ atom of adenine [50]. Villa et al. reported that insulin stimulation increased the methyltransferase WTAP expression and promoted MAT2A and SAM production, finally facilitating N^6^-methylation of mRNA [51]. These provided evidence that SAM catalyzed by MAT2A participated in the formation of m^6^A RNA methylation. Similarly, in this study, MAT2A expression was also significantly increased in the LPS group with high m^6^A levels, and this concurred with the change in ROS and HIF-1α levels. Therefore, we speculated that high ROS levels induced by LPS in the liver promoted HIF-1α expression, and subsequently increased the MAT2A level. The change of ROS, HIF-1α, and MAT2A may contribute to the increase of m^6^A RNA methylation, which plays a critical role in the activation of NOD1/NF-κB signaling pathway.

It is well-known that the NOD1/NF-κB signaling pathway plays a vital role in host defense and proinflammatory response. Many studies implied that NOD1 gene expression was regulated by various epigenetic modifications that occurred in DNA, RNA, and proteins. Wang et al. reported that the increase of DNA methylation and chromatin compaction in the NOD1 promoter region resulted in a decline in NOD1 gene expression [52]. Martins et al. demonstrated that the acetylation of histone H3 was closely associated with the activation of NOD1 signaling in human oral epithelial cells [53]. Furthermore, miR-495 [54], miR-147, and miR-217 [55] directly targeted NOD1 through 3′UTR sequence binding, followed by regulating its gene expression. As an epitranscriptomic marker, m^6^A RNA methylation is an emerging layer of posttranscriptional gene regulation. However, little information is available describing the link between m^6^A modification and NOD1 gene expression. In this study, we identified a quantitative difference in m^6^A peaks in the CON and LPS groups by using MeRIP-seq, implying that m^6^A methylation patterns visually changed after LPS stimulation. Moreover, gene enrichment analysis revealed that m^6^A methylated genes in the LPS group were mostly distributed in pathways associated with immune and inflammation, such as the NF-κB signaling pathway. This observation is in line with Guo et al. reports [41], in which differential methylated transcripts were significantly enriched in pathways closely related to immune response in the liver of LPS-treated chicken. Notably, we found that among those methylated genes, the expression of NOD1 was upregulated, and m^6^A modification distributed on mRNAs in the NOD1/NF-κB pathway were significantly altered under LPS administration, suggesting that m^6^A RNA methylation may be involved in gene expression of the NOD1/NF-κB pathway. Wang et al. reported that knockdown of METTL3 upregulated the NOD1 signaling pathway by affecting mRNA degradation in a YTHDF1- and YTHDF2-dependent manner [56]. Our data in HepG2 cells are in agreement with Wang et al. observations, in that the knockdown of METTL3 significantly upregulated the expression of NOD1 mRNA. Besides, we noted that the NF-κB p65 mRNA level was also significantly increased by knocking down METTL3, in accordance with increased NF-κB p65 phosphorylation in METTL3-deficient macrophages [57] and reduced p65 phosphorylation in METTL3-overexpressed pTHP-1 cells [58]. Similarly, overexpression of FTO also enhanced the mRNA levels of NOD1 and NF-κB p65 in HepG2 cells, which may be due to the increased mRNA stability caused by decreased m^6^A modification. Taken together, our results indicated that m^6^A RNA methylation enabled regulate the NOD1/NF-κB signaling pathway in piglets challenged with LPS.

## 5. Conclusions

**Figure 7:**
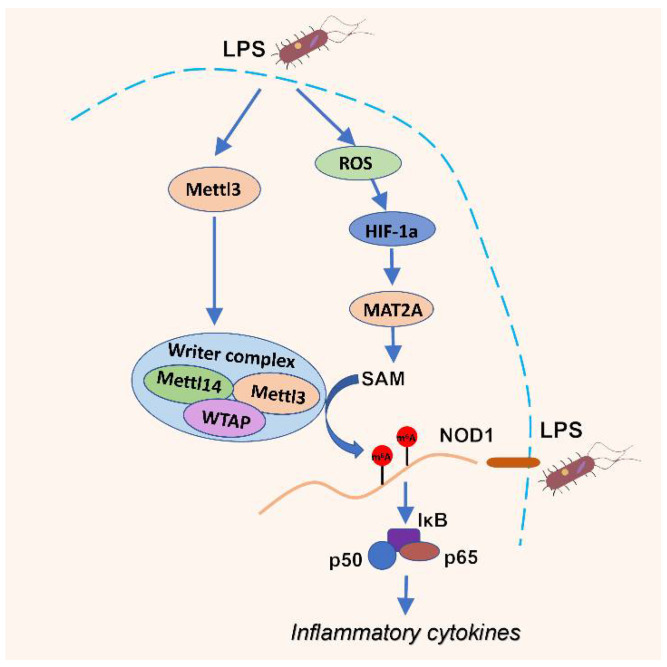
The proposed mechanism for the regulation of m^6^A RNA methylation in the NOD1/ NF-κB signaling activation in the liver of piglets challenged with LPS.

In conclusion, our experiment elucidated m^6^A RNA methylation is critical in hepatic inflammation and injury induced by LPS through regulating the activation of the NOD1/NF-κB signaling pathway. Aside from this, our study firstly provided the possibility that the increase of ROS, HIF-1α, and MAT2A expression induced by LPS contributed to elevate m^6^A RNA methylation in the liver of piglets (Figure 7). These findings in the current study implied that NOD1 and m^6^A RNA methylation could be novel viewpoints to explore and deal with immune stress-induced liver damage in piglets. Further work should determine the precise mechanisms of m^6^A RNA methylation regulating the fate of NOD1 by functional readers.

## Figures and Tables

**Table 1:** Primer sequences used for quantitative real-time PCR.

Gene Name ^1^		Primer Sequence (5′-3′)	Gene Bank Number
GAPDH	Forward	CGTCCCTGAGACACGATGGT	AF017079.1
	Reverse	GCCTTGACTGTGCCGTGGAAT	
METTL3	Forward	TGAGGCTCCTGGAAGCAAAG	XM_003128580.5
	Reverse	TCTGTCAGGGTCCCATAGGG	
METTL14	Forward	GTGGTTCTGGGGAGGGATTG	XM_003129231.6
	Reverse	GAAGTCCCCGTCTGTGCTAC	
FTO	Forward	CCCCAGAAAATGCCGTACCT	KM232950.1
	Reverse	ACCAGGGGTCTCTATGTCCC	
ALKBH5	Forward	CGTGTCCGTGTCCTTCTTCA	XM_021067995.1
	Reverse	AGGATGATGACAGCTCTGCG	
YTHDF1	Forward	GCACCGCTCCATCAAGTACT	MN606020.1
	Reverse	GCTGAAGAGCAGGTAGACGG	
YTHDF2	Forward	CCAAGGGATGGCAGCACTAA	XM_005665152.3
	Reverse	TTTGCCACAGGACCCTTGTT	
YTHDF3	Forward	GAGCAAGGCATGACTGGACT	MN606021.1
	Reverse	CTGGGGGCACACTATTGGTT	
IL-4	Forward	ACACGACGGAGAAGGAAACC	NM_214123.1
	Reverse	GTTCCTGTCAAGTCCGCTCA	
IL-10	Forward	TCGGCCCAGTGAAGAGTTTC	NM_214041.1
	Reverse	CGGCATTACGTCTTCCAGGT	
TNF-α	Forward	TCCAATGGCAGAGTGGGTATG	NM_214022.1
	Reverse	AGCTGGTTGTCTTTCAGCTTCAC	
IL-1β	Forward	GCTGATGGCCCCAAAGAGAT	NM_001302388.2
	Reverse	TGCCACAATCACAGACACCA	
IL-6	Forward	AAATGTCGAGGCTGTGCAGA	NM_214399.1
	Reverse	TCCACTCGTTCTGTGACTGC	
TLR4	Forward	TCAGTTCTCACCTTCCTCCTG	GQ503242.1
	Reverse	GTTCATTCCTCACCCAGTCTTC	
MyD88	Forward	GATGGTAGCGGTTGTCTCTGAT	AB292176.1
	Reverse	GATGCTGGGGAACTCTTTCTTC	
NOD1	Forward	CTGTCGTCAACACCGATCCA	AB187219.1
	Reverse	CCAGTTGGTGACGCAGCTT	
RIPK2	Forward	CAGTGTCCAGTAAATCGCAGTTG	XM_003355027.1
	Reverse	CAGGCTTCCGTCATCTGGTT	
NFKBIA	Forward	TGTTGGTGTCTTTGGGTGCT	NM_001005150.1
	Reverse	GACATCAGCCCCACACTTCA	
NF-κB p65	Forward	TACTGATGAGGACCTGGGGG	NM_001114281.1
	Reverse	ATACACCCTGGTTCAGCAGC	
HIF-1α	Forward	AGCCAGATGATCGTGCAACT	NM_001123124.1
	Reverse	CCATTGATTGCCCCAGGAGT	
MAT2A	Forward	GCACACAAGCTCAATGCCAA	NM_001167650.1
	Reverse	ACTCTGATGGGAAGCACAGC	
iNOS	Forward	CCAGGCAATGGAGAGAAACT	NM_001143690.1
	Reverse	CCGAACACAGCATACCTGAA	
CD86	Forward	TGGTGCTGCCTCCTTGAAAA	L76099.1
	Reverse	GGACACAGACGATGCTCACA	
IL-12	Forward	GGACTGCGTCTTCACTTCCA	AF330213.1
	Reverse	TGGAGTTAGCTGCAGACACG	
CD206	Forward	GCCCAGACTGAAGACAGCAT	JN989538.1
	Reverse	GGCATCTACCAGGCAGTTGT	

^1^ GAPDH, glyceraldehyde-3-phosphate dehydrogenase; METTL3, methyltransferase like 3; METTL14, methyltransferase like 14; FTO, fat mass and obesity-associated protein; ALKBH5, α-ketoglutarate-dependent dioxygenase alkB homolog 5; YTHDF1, YTH domain protein 1; YTHDF2, YTH domain protein 2; YTHDF3, YTH domain protein 3; IL-4, interleukin-4; IL-10, interleukin-10; TNF-α, tumor necrosis factor alpha; IL-1β, interleukin-1β; IL-6, interleukin-6; TLR4, toll-like receptor 4; MyD88, myeloid differentiation primary response gene 88; NOD1, nucleotide binding oligomerization domain containing 1; RIPK2, receptor interacting Serine/Threonine kinase 2; NFKBIA, nuclear factor of kappa light polypeptide gene enhancer in B cells inhibitor, alpha; NF-κB p65, nuclear transcription factor kappa p65 protein; HIF-1α, hypoxic inducible fator-1 alpha; MAT2A, methionine adenosyltransferase Ⅱ alpha; iNOS, inducible nitric oxide synthase; CD86, clusters of differentiation 86; IL-12, interleukin-12; CD206, clusters of differentiation 206.

## Data Availability

According to the journal guidelines, the accession number for the MeRIP-seq data reported in this paper is GEO: GSE 209858.
